# Single Lung Transplant for Secondary Pulmonary Hypertension: The Right Option for the Right Patient

**DOI:** 10.3390/jcm12216789

**Published:** 2023-10-27

**Authors:** Brian Housman, Daniel Laskey, Gbalekan Dawodu, Scott Scheinin

**Affiliations:** Department of Thoracic Surgery, Icahn School of Medicine at Mount Sinai, Mount Sinai Health System, New York, NY 10029, USA; daniel.laskey@mountsinai.org (D.L.); gbalekan.dawodu@mountsinai.org (G.D.); scott.scheinin@mountsinai.org (S.S.)

**Keywords:** single lung transplant, double lung transplant, pulmonary hypertension, rejection, complications, extracorporeal membranous oxygenation

## Abstract

**Introduction**: The optimal treatment for Secondary Pulmonary Hypertension from End-Stage Lung Disease remains controversial. Double Lung Transplantation is widely regarded as the treatment of choice as it eliminates all diseased parenchyma and introduces a large volume of physiologically normal allograft. By comparison, the role of single lung transplantation for pulmonary hypertension (PAH) is less clear. The remaining diseased lung will limit clinical improvements and permit downstream sequelae; including residual cough, recurrent infection, and continued pulmonary hypertension. But not every patient can undergo DLT. Advanced age, frailty, co-morbid conditions, and limited availability of organs will all affect surgical candidacy and can offset the benefits of double lung procedures. Studies that compare SLT and DLT do not commonly explore the utility of single lung procedures even though multiple theoretical advantages exist; including reduced waiting times, less waitlist mortality, fewer surgical complications, and lower operative mortality. Worse, multiple forms of publication and selection bias may favor DLT in registry-based studies. In this review, we present the prevailing literature on single and double lung transplants in patients with secondary pulmonary hypertension and clarify the potential utility of these procedures. **Materials and Methods:** A PubMed search for English-language articles exploring single and double lung transplants in the setting of secondary pulmonary hypertension was conducted from 1990 to 2023. Key words included “single lung transplant”, “double lung transplant”, “pulmonary hypertension”, “rejection”, “complications”, “extracorporeal membranous oxygenation”, “death”, and all appropriate Boolean operators. We prioritized research from retrospective studies that evaluated clinical outcomes from single centers. **Conclusions:** The question is not whether DLT is better at resolving lung disease; instead, we must ask if SLT is an acceptable form of therapy in a select group of high-risk patients. Further research should focus on how best to identify recipients that may benefit from each type of procedure, and the clinical utility of perioperative VA ECMO.

## 1. Introduction

The optimal treatment for Secondary Pulmonary Hypertension (SPH) from End-Stage Lung Disease (ESLD) remains controversial. Double Lung Transplantation (DLT) is widely regarded as the treatment of choice as it eliminates all diseased parenchyma and introduces a large volume of physiologically normal allograft. In practice, this normalizes functional capacity, pulmonary vascular resistance (PVR), and right ventricular systolic function [1,2,3]. Additionally, though contentious in the literature, DLT recipients are reported to be more resistant to the effects of reflux, bronchiolitis obliterans, and both acute and chronic rejection [3,4,5,6,7,8,9]. However, DLT is also associated with higher rates of perioperative complications, including bleeding, bronchial dehiscence, dialysis, increased length of stay, and early post-operative death [4,10].

By comparison, the role of single lung transplantation for pulmonary hypertension (PAH) is less clear [11]. The remaining diseased lung will limit clinical improvements and permit downstream sequelae; including residual cough, recurrent infection, and continued pulmonary hypertension [4]. In addition, lower vascular resistance in the transplanted lung means greater blood volumes will preferentially flow into the allograft. This will increase rates of endothelial sheer stress, hemodynamic instability, primary graft dysfunction, and Ventilation/Perfusion (V/Q) mismatches, which all theoretically contribute to earlier presentations of chronic lung allograft dysfunction (CLAD) [11,12,13].

But not every patient can undergo DLT. Advanced age, frailty, co-morbid conditions, and the limited availability of organs will all affect surgical candidacy and can offset the benefits of double lung procedures [1,6,9,10,11]. Studies that compare SLT and DLT do not commonly explore the utility of single lung procedures even though multiple theoretical advantages exist; including reduced waiting times, less waitlist mortality, fewer surgical complications, and lower operative mortality [6,9]. Worse, multiple forms of publication and selection bias may favor DLT in registry-based studies.

Because of this multitude of factors, surgeon judgement plays a central role in safe operative choices for transplant patients. This is likely why a prospective randomized trial has never been performed and the best transplant procedure for PAH may never be codified [1,6].

In this review, we present the prevailing literature on single and double lung transplants in patients with secondary pulmonary hypertension and clarify the potential utility of these procedures.

## 2. Materials and Methods

A PubMed search for English-language articles exploring single and double lung transplants in the setting of secondary pulmonary hypertension was conducted from 1990 to 2023. Key words included “single lung transplant”, “double lung transplant”, “pulmonary hypertension”, “rejection”, “complications”, “extracorporeal membranous oxygenation”, “death”, and all appropriate Boolean operators. We prioritized research from retrospective studies that evaluated clinical outcomes from single centers. Studies that discussed risk factors, mechanisms, and outcomes were also included from multicenter trials, retrospective database research, and other review articles. The guidelines, original works, and foundational studies from professional societies and individual leaders in the field were also reviewed. Only articles agreed upon by all authors were included.

### 2.1. Double Lung Transplant as the Standard of Care

Double Lung Transplant for pulmonary hypertension began to replace Heart–Lung Transplant (HLT) from the late 1980s to early 2000s [11,13]. In 2004, Kasimir et al. reported durable improvements in mean pulmonary artery pressure (mPAP) and right ventricular ejection fraction immediately after surgery and persistent for months [11,14]. Survival data at the same time showed that DLT was equivalent or better than HLT and, by 2010, DLT was considered the standard of care for PAH [15,16].

Multiple studies have since been performed that support the use of DLT as a treatment for secondary pulmonary hypertension. As early as 1994, a team led by Dr. Ko Bando investigated the long-term outcomes in patients treated with SLT, DLT, and HLT for pulmonary hypertension [13]. They examined 24 patients undergoing SLT (n = 11), DLT (n = 22), and HLT (n = 24) performed between 1989 and 1993 for primary pulmonary hypertension and Eisenmenger’s syndrome [13]. Patients underwent HLT when they had an ejection fraction <35%, coronary artery disease, or Eisenmenger’s syndrome from congenital heart disease. All other patients underwent SLT or DLT depending on donor availability [13].

They observed that following SLT, patients required longer periods of mechanical ventilation and longer stays in the ICU [13]. The authors stated that early mortality following the three procedures was similar, but there were only two deaths in the SLT group and seven combined in the BLT/HLT groups. Of note, both deaths following SLT were graft-related, compared to only one of seven following bilateral or combined procedures [13]. The remainder were due to bleeding, infection, and technical complications from the surgery itself [13]. As a result, graft-related mortality was found to be significantly higher following SLT compared to BLT or HLT [13].

The authors found that all patients experienced a decrease in mPAP. However, the pressures were significantly higher in the SLT group compared to DLT or HLT. The cardiac index (CI) significantly only improved in patients who underwent DLT or HLT. There was also a significant decrease in pulmonary vascular resistance following DLT and HLT, but not SLT [13].

The authors also found that while perfusion to the transplanted allograft substantially increased, ventilation did not change; resulting in a significant V/Q mismatch. This effect was also observed by Levine and colleagues in 1990 [17].

Interestingly, the 1-year survival following SLT was lower than BLT and HLT, but the difference was not statistically significant [13]. As the original purpose of the study was to investigate the long-term outcomes of these groups, the absence of a meaningful change in long-term mortality is a compelling finding.

Nasir and colleagues published a study utilizing the OPTN/UNOS Standard Transplant and Analysis Research Registry [18]. They examined 12,392 patients between 2005 and 2015 comparing patients with mean pulmonary artery pressures (mPAP) below and above 40 mm Hg who were undergoing both SLT and DLT.

In patients undergoing SLT, 5-year overall survival (OS) was lower in patients with mPAP > 40 compared to patients with mPAP < 40 (43.9% v. 48.2%, *p* = 0.007). In patients undergoing BLT, there was no difference in 5-year survival even between patients with low and high mPAP (57.3% v. 55.8%, *p* = 0.15) [18]. They also found that mPAP > 40 was an independent predictor of mortality in SLT with a hazard ratio of 1.31. By contrast, patients with elevated mPAP in DLT were not found to have an increased risk of death (HR 1.04, *p* = 0.48) [18].

One potential weakness of the paper is that the authors did not consider intraoperative conditions, including the use of perioperative ECMO. Of course, the study was a retrospective database review and was likely limited in terms of data collection.

Another study performed by Villavicencio examined BLT versus SLT for pulmonary fibrosis [19]. They specifically questioned whether bilateral lung transplants would benefit older patients (≥70 years) with high lung allocation scores [19]. They queried the UNOS database between 1987 and 2015 for all recipients of lung transplants for pulmonary fibrosis and found 9191 patients [19]. The 10-year survival rate was 55% for BLT and 32% for SLT. Bilateral lung transplant recipients had improved survival across all lung allocation scores (examined at <45, ≥45, ≥60, ≥75) and all age cutoffs except patients ≥70 years [19]. Bilateral patients also had improved survival at all evaluated pulmonary artery pressures (<25, ≥25, ≥30, ≥40 mm Hg). For single lung recipients, mPAP ≥30 and a lung allocation score ≥45 had decreased survival. Bilateral lung recipients’ mPAP and lung allocation score did not affect survival [19].

Antończyk published a single-center experience for patients undergoing single and double lung transplants [20]. They evaluated 128 patients who underwent transplants from 2004 to 2017 for COPD, cystic fibrosis, primary pulmonary hypertension, and interstitial lung disease. They found that the overall 5-year survival for BLT was 75% and SLT was 51% [20]. For patients with primary PAH, the 5-year survival for BLT was 84% and SLT was 51%. While they did not specify patients with secondary PAH, the 5-year survival in COPD for BLT was 82% and SLT was 62%.

The authors specifically noted that patients who underwent SLT were significantly older than patients who underwent DLT 50 v. 35 years, *p* < 0.001) and had a higher body mass index (21.93 v. 19.6, *p* < 0.001) [20]. Interestingly, double lung recipients also had a lower median forced expiratory volume after 1 s (FEV_1_) at 24% compared to 30% of expected volumes (*p* < 0.001) [20]. Notably, the patients with idiopathic pulmonary arterial hypertension did not receive perioperative support with ECMO for left heart conditioning [20].

Recently, in 2022, Hansmann and colleagues described the full recovery of right ventricular function in children following bilateral lung transplants [21]. They evaluated 15 children (ages 1.9 to 17.6 years) who consecutively underwent bilateral lung transplants without heart transplants [21]. They found that after DLT, right ventricular volumes and systolic function completely normalized, even in patients with severe right heart failure (RVEF < 40%) [21]. In patients with severe PAH, there was decreased radial and circumferential strain and reduced longitudinal strain of the right ventricle, all of which significantly improved following surgery [21].

Since 2007, DLT has been reported to be an independent protector factor against mortality, with a hazard ratio of 0.737 [22]. At 10 years, 22% more DLT recipients are alive with a mean survival of 3.2 years better than for SLT recipients [22]. In fact, the 2007 report commissioned by the International Society of Heart and Lung Transplantation (ISHLT) described the superiority of DLT after controlling for 19 variables [22].

### 2.2. Single Lung Transplant May Be a Viable Option

Despite many such reports in the literature, improvements in mPAP following SLT have been described since the early 1990s [17,23,24]. A team in San Antonio led by Dr. Stephanie Levine showed “marked” decreases in mPAP and PVR with improvements in cardiac output in three patients following SLT [17]. The changes in mPAP from pre-SLT to post-surgery were (1) 70 to 28 mm Hg, (2) 47 to 17 mm Hg, and (3) 62 to 17 mm Hg [17]. For cardiac output and the cardiac index, the changes were (1) CO 2.1 to 5.3 LPM, CI 1.5 to 3.8 L/min/m^2^, (2) CO 2.5 to 6.6 LPM, CI 1.7 to 4.4 L/min/m^2^, and (3) CO 2.9 to 5.5 LPM, CI 1.6 to 3.1 L/min/m^2^. Interestingly, there were small and inconsistent improvements in both spirometry and DLCO [17]. Again, this appears to suggest that clinical improvements following SLT in PAH are more attributable to restored perfusion instead of ventilation [13,17].

In 1991, Maurer and colleagues showed improved right ventricular function and pulmonary pressures in six patients with end-stage PAH after SLT [23]. In 1990, the first report of SLT for Eisenmenger’s Syndrome was published [25]. In 1992, Pasque et al. published the outcomes of nine patients with end-stage PAH [24]. They described early decreases in pulmonary pressures and restored right ventricular function from NYHA Class III or IV to Class I with an 89% survival at 1 year [24]. Notably, the authors described routine intraoperative use of cardiopulmonary bypass [24].

In the early era, these studies served more as a proof-of-concept. They verified, in human subjects, the long-held belief that SLT can improve hemodynamics in patients with pulmonary hypertension. Unfortunately, they lacked the reliability of long-term outcomes or survival statistics.

However, survival advantages are not always observed following DLT, and when they are, a meaningful benefit is often only proven after 1 year [10,19,26,27,28]. Villavicencio and colleagues showed that, for patients < 60 years, the survival advantage of DLT is nearly immediately apparent after surgery [19]. However, for patients > 60, the advantage is not observed until 10 months. For patients older than 65, it is not until 18 months, and for patients > 70, there is no observed benefit from DLT over SLT [19]. Propensity-matched analysis showed a survival advantage of DLT over SLT, but not until almost 1.5 years [19]. By contrast, a paper by Meyers et al. showed that even for patients < 60 years, there was a survival benefit for SLT over DLT [29].

In 1994, Dr. Bando and his team investigated patients with pulmonary hypertension treated with SLT (n = 11), BLT (n = 22) and HLT (n = 24) [13]. They found that 1-month and 3-month survival was similar among the three groups. The 1-year survival was lower in SLT, but was not statistically significant [13].

Fitton and colleagues examined 45 patients with SPH who underwent both SLT and DLT [2]. There were 28 patients with low PAH (mPAP 30–40 mm Hg) and 17 patients with high PAH (mPAP ≥ 40 mm Hg) [2]. These were compared to 42 lung transplant patients with normal pulmonary pressures who served as the controls. Unsurprisingly, there were significant differences in pre-operative oxygen dependence, A-a gradients, and the use of cardiopulmonary bypass [2]. However, there was no significant difference in survival at 1, 2, and 4 years between the three groups. (Low PAH—85%, 72%, and 72%, High PAH—82%, 73%, 73%, Controls—76%, 58%, and 49%) [2]. Interestingly, when comparing the 45 patients with PAH by procedure, survival was actually worse in the BLT group [2]. At 1, 2, and 4 years, survival for SLT (n = 23) was 82%, 82%, and 82%, and for DLT (n = 22) it was 87%, 62%, and 62% [2].

Thabut and colleagues evaluated 3327 patients who underwent transplantation between 1987 and 2009 [26]. They used multiple methods of risk adjustment including propensity-based matching and multivariable regression. Despite these efforts, they found no difference in long-term survival between patients undergoing SLT and DLT [26].

Chauhan and colleagues reviewed the UNOS registry from 2001 to 2009, looking at post-transplant survival in patients with idiopathic pulmonary fibrosis (IPF) [30]. They examined patients who were listed for both single and double lungs. Of the 1001 recipients, 434 (43%) underwent SLT and 566 (57%) underwent BLT [30]. Post-transplant survival was reported in graft survival years. There were 2722.5 years at risk with a median graft survival of 5.31 years [30]. They found no difference in graft survival between SLT and DLT [30]. Survival at 3 months for SLT was 93.30% and for DLT it was 89.91%. At 1 year, SLT was 83.27% and DLT was 80.26%. At 5 years, SLT was 51.68% and DLT was 53.43% [30]. They also found substantial differences in co-morbidities, pulmonary function tests, baseline disease severity, and overall functional status [30].

Recently, Sunagawa and colleagues at Temple University examined all patients undergoing SLT lung transplants at their institution from 2017 to 2019 [9]. They stratified PAH as severe (mPAP > 40 mm Hg) or mild (mPAP 25–40 mm Hg), and patients without PAH (mPAP < 25 mm Hg) were used as the controls. Since the study was institutional, the authors were able to consider pre-operative, intra-operative, and post-operative patient characteristics, unlike most retrospective analyses. Of note, all patients with primary pulmonary hypertension were excluded, since all patients underwent DLT.

A total of 318 patients underwent SLT during the study period. A total of 59 patients had severe PAH (18.5%), 217 had mild PAH (68.2%), and another 42 patients had no PAH (13.2%). The mPAP in the severe category was 44 mm Hg, mild was 31 mm Hg, and normal was 22 mm Hg [9].

Pre-operatively, in addition to pulmonary pressure and vascular resistance, body mass index (BMI) was significantly higher in PAH groups compared to the control group. Of note, two patients in the mild PAH group were on veno-venous (VV) extracorporeal membranous oxygenation (ECMO) before transplantation [9].

Intra-operatively, the use of mechanical circulatory support (MCS), including both cardiopulmonary bypass (CPB) and ECMO, was higher in the severe group than the mild group, and both were higher than controls (37.3% v. 10.3% v. 4.8%) [9]. One patient in the severe group and three in the mild group (including the two patients who were pre-operatively placed on VV ECMO) were transported out of the operating room on ECMO. The authors noted that all cases were due to primary graft dysfunction (PGD) [9].

Between the three groups, there were no differences in terms of 1-year survival (*p* = 0.58), 3-year survival (*p* = 0.37), 30-day mortality, 90-day mortality, patients who remained intubated at 72 h (*p* = 0.13) or 120 h (*p* = 0.79), the rate of reintubation (*p* = 0.17), tracheostomy placement (*p* = 0.69), PGD requiring ECMO (*p* = 0.25), or the use of hemodialysis (*p* = 0.37) [9]. Interestingly, the two patients with severe PAH who required dialysis had their SLT performed without MCS. None of the patients with severe PAH who were supported with intra-operative ECMO (10 patients) or CPB (12 patients) required post-operative hemodialysis [9].

There was a trend toward a longer hospital stay in patients with severe PAH. However, the finding was not statistically significant, and the median was actually lower in both PAH groups (Normal 16 (Range 11–21), Mild 14 (Range 11–20), and Severe 14 (11–27), *p* = 0.38) [9].

Though not statistically significant (*p* = 0.25), there was another noteworthy trend in the number of patients requiring ECMO for PGD; 0 of 42 in the normal group (0%), 12 of 217 in the mild group (5.5%), and 2 of 59 in the severe group (3.4%). However, it stands to reason that patients with increasingly severe diseases are more likely to require both intra-operative and post-operative mechanical support.

There were no significant differences in 1-year (severe 93.2% v. mild 89.4% v. normal 92.9%, *p* = 0.58) or 3-year survival (severe 79% v. mild 75.1%, v. normal 65.4%, *p* = 0.37). Interestingly, patients with severe PAH had a trend toward the best outcomes of all three groups [9].

Unfortunately, since DLT is considered the standard of care for PAH, few studies in the literature compare outcomes with SLT. Nevertheless, several studies offer valuable findings regarding the limitations of DLT in other select populations.

Gulack and colleagues at Duke University examined 1564 patients from 2005 to 2013 in the United Network for Organ Sharing (UNOS) database [10]. They compared survival by transplant between SLT and DLT. Following propensity-matched analysis, they found that DLT had a 5-year survival advantage (48.7% v. 35.2%, *p* < 0.01) even in patients older than 65 years. However, this benefit was only observed beyond 1 year. DLT patients had a higher rate of airway dehiscence (3.9 v. 1.0%, *p* < 0.01), dialysis (9.2% v. 2.7%, *p* < 0.01), and longer hospital length of stay (19 days v. 13 days, *p* < 0.01). Most importantly, in patients with a low functional status, DLT patients had higher early morbidity and mortality [10]. It is likely that higher rates of perioperative complications associated with DLT could only be survived by patients with the clinical resiliency to tolerate them.

Meyer and colleagues investigated survival by comparing technique and age; 41–50, 51–60, and 61–70 [5]. From 1991 to 1997, there were 2260 lung transplants (1835 SLT and 425 Sequential BLT). Interestingly, they found better short- and long-term survival in younger patients (30–49 years) undergoing SLT compared to similar patients undergoing BLT [5]. Among younger patients (<60 years), there was an advantage in receiving BLT with a diminishing advantage as the patient age approached 60 [5]. Beyond 60 years, the trend abruptly reversed and survival associated with SLT was markedly higher [5]. While these two studies did not specifically describe PAH patients, they did describe the same phenomenon suggested by the Temple group: age will significantly affect organ availability, and the capacity of the patient to tolerate surgery.

Even Bando and colleagues, in 1994, described improvements in mPAP and PVR following SLT that appeared to be comparable to DLT and HLT [13]. While graft-related mortality was found to be higher following SLT, 85% of early deaths following BLT or HLT were technical or related to surgical recovery [13]. And this was in a study that was supposed to codify the superiority of DLT.

### 2.3. The Use of ECMO

There are some studies that suggest perioperative ECMO may offset some of the risks of transplant for PAH. The phenomenon of circulatory failure following lung transplantation for long-standing PAH is well described. Chronic pulmonary vascular resistance results in right ventricular hypertrophy and dysfunction. This results in decreased right ventricular cardiac output that in turn decreases preload and compliance in a chronically underfilled left ventricle. Following transplantation, the pulmonary hypertension resolves, right ventricular afterload decreases, and the left ventricle is exposed to a “normal” preload volume. As a result, patients experience left ventricular failure due to diastolic dysfunction combined with increased end-diastolic volume [12].

This phenomenon is compounded by the effects of allograft that is already predisposed to endothelial leak. All transplanted lungs experience some degree of ischemia—reperfusion injury; though pulmonary hypertension is also an additional independent risk factor [31,32]. The resulting inflammation causes endothelial leak, which is worsened by endothelial shear forces from the hypertrophic right ventricle, and results in interstitial edema and alveolar flooding. At the same time, elevated hydrostatic forces from increased left ventricular preload also contribute to elevated intrapulmonary pressures and facilitate third spacing. The magnitude of edema and alveolar flooding is therefore related to the transpulmonary gradient, or the amount of blood physically moving through the pulmonary vasculature under these conditions [11,31].

Since blood will preferentially flow to the new lung with a normal PVR, this effect may be worse after SLT. A single transplanted lung may therefore experience a higher degree of vascular strain than either individual graft following a bilateral transplant. In fact, this may explain why SLT is sometimes suggested as an independent risk for PGD [12,13].

Common strategies to avoid acute left ventricular failure include inotropic medications to assist with myocardial ejection and limiting left ventricular preload by reducing transpulmonary blood flow. This is routinely accomplished with fluid restriction and diuresis, but several studies have also shown the benefit of prolonged perioperative VA ECMO [11,33].

Salman and colleagues examined all patients undergoing lung transplants between 2010 and 2016 [34]. Over this time, 717 patients received lung transplants, 38 of which were for severe pulmonary hypertension. All 38 underwent BLT with VA ECMO support through the groin. Interestingly, they performed sternum-sparing bilateral thoracotomy in 36 of these patients [34]. Patients were extubated after a median duration of 2 days, and awake VA ECMO was continued for 5 days following surgery [34]. They found no differences in survival after 3 months, 1 year, and 5 years between the 38 patients with severe PAH and the other transplant recipients (*p* = 0.45) [34]. Furthermore, echocardiographic data were reviewed, which showed that ECMO resulted in shorter post-operative intubation, reduced post-operative mortality, and a survival benefit that persisted up to 5 years [34].

In 2018, Moser found that post-operative VA ECMO for pulmonary hypertension improves both short– and long–term survival [33]. The study examined 41 patients undergoing bilateral lung transplants for idiopathic pulmonary hypertension. Following intraoperative support, VA ECMO was post-operatively continued for a median time of 2.5 days. This group was compared to another 31 patients who underwent transplantation without post-operative ECMO support. The survival rates appeared to strongly favor the group who received continued ECMO. Survival at 90 days was 92.7% v. 83.9%, at 1 year it was 90.2% v. 77.4%, at 3 years it was 87.4% v. 77.4%, and at 5 years it was 87.4% v. 77.4% [33]. These findings did not achieve statistical significance (*p* = 0.189) but that is likely because the study had an underpowered population.

A study by Kortchinsky et al. examined the short- and long-term outcomes in patients requiring post-operative VA ECMO [12]. They examined 93 patients with pulmonary hypertension who underwent heart and lung transplants (n = 29), and bilateral lung transplants (n = 64). ECMO was started or continued at the end of the transplant for pre-operative mPAP > 40 mm Hg, Grade III primary graft dysfunction, pulmonary edema with hemodynamic failure, rapid hemodynamic deterioration (defined as a need for high-dose catecholamine vasopressors), and deoxygenation (defined at saturation < 95% with FiO_2_ at 100% and Nitric Oxide) [12].

Of the ninety-three total patients, twenty-eight patients required ECMO; six in the heart and lung group and twenty-two in the bilateral lung group. ECMO was started in all patients within 6 h of transplantation and kept for an average of 3 days (2.0–8.5 days). Successful weaning occurred in 24 of the 28 patients [12]. Patients who did not require ECMO had a higher survival at 30 days (95% v. 78.5%, *p* = 0.02) and at 1 year (83% v. 64%, *p* = 0.005). They also found that patients undergoing ECMO had more frequent infections (79% v. 48%, *p* = 0.0006), and more frequent bleeding events (43% v. 17%, *p* = 0.008) [12].

The authors concluded that ECMO was an effective treatment of PAH, PGD, hemodynamic compromise, and hypoxia, but was associated with higher rates of infection and bleeding [12]. Unfortunately, the study was unable to delineate the difference between patients who underwent planned peri-operative ECMO versus patients who required support following emergent clinical decline.

This suggests that some of the risks of lung transplantation for PAH are ameliorated using perioperative ECMO support. Gradual myocardial conditioning through a reduction in transpulmonary flow may offset the hemodynamic strain of a rapidly normalized PVR. Since a single graft following SLT may experience greater vascular strain than either individual graft following DLT, this effect may be even more compelling for SLT. Future research comparing SLT and DLT should explore the value of mechanical support to facilitate safe single lung procedures.

### 2.4. Bias in the Literature

The lack of consensus in the literature may have a simple explanation: single and double lung recipients may not be comparable groups. Patients undergoing SLT are likely to be older, frailer, have lower functional status, worse renal function, worse pulmonary function, elevated diastolic pressures, stroke history, severe disease requiring ICU treatment, and accept lungs from older donors [2,9,13,19,20,30]. This population is less likely to tolerate bilateral surgery or longer waiting times, and because of concomitant medical conditions, will have more complex perioperative recovery. Additionally, with older donors, long-term outcomes will likely be affected even following successful surgery. Even if a survival benefit exists for DLT, higher-risk patients with pulmonary hypertension may still experience a marked benefit from SLT due to lower procedural risk and shorter waiting times.

As a result, publications comparing single and double lung recipients may not be completely reliable. Studies may be saturated by publication and selection biases caused by patient selection, faulty data gathering, and surgeon factors in the following ways:

(1) There is an inherent bias that will favor the outcomes of younger and healthier patients who tend to receive DLT over SLT [9,19,20].
(2) There is substantial variability in the willingness to accept a lung donor at both a surgeon level and an institutional level [28,30].
(3) Patients who are concomitantly listed for DLT or SLT and undergo SLT due to clinical decline may be included in the SLT group [30].
(4) Organ availability is not uniform throughout the United States. Different centers will be affected by variability in donor offers and recipient waiting times [35].
(5) Patient characteristics are not uniform throughout the United States. Clusters of patients with higher or lower risk features can affect clinical practice and statistical evaluation [9].

Though propensity-matched analysis can be helpful, the premise that SLT and DLT recipients are comparable may be intrinsically flawed. There is significant variation in surgical choice, available resources, institutional policies, and the clinical features of patient populations. [6,9] Recipients of single lungs are frequently older, less robust, and continue to endure the effects of pre-existing illnesses in their retained lung. This will have both ventilatory and cardiovascular consequences that require ongoing treatment in addition to the need for post-transplant care. Additionally, most studies advocate for the superiority of DLT without regard for the underlying lung disease [9]. Little effort is made to examine the confounding effects of these variables or the clear and notable advantages of SLT; including reducing post-operative morbidity, shorter times on the waitlist, earlier recovery, and increased candidacy for frail and elderly patients [1,9].

Waitlist mortality is highest in patients older than 65 years and patients with restrictive lung disease; those who are also the most likely to have secondary pulmonary hypertension [36]. The Temple group described that the number of patients over 70 years on their waitlist is three times the national average (31.8% v. 12.0%), and two times the average of the regional network (31.8 v. 16.2) [9]. Theoretical or not, the benefit of DLT has to be weighed against increased perioperative risk and the survival disadvantage of longer waiting times; especially in regions with limited organ availability.

Unfortunately, an alternative research method may not be possible. Prospective studies would need to be conducted involving matched cohorts with similar ages and clinical conditions. Patients would need to be randomized into single and double lung treatment arms, which—due to the limited organ availability, dynamic clinical changes, and individual surgeon judgment—would be impractical and likely unethical.

The recent work by Sunagawa and colleagues from Temple University is one of the best efforts at retrospective comparison, especially in a high-risk population [9]. Perhaps future efforts could be made to group outcomes by patient severity as well as procedure type.

## 3. Conclusions

With only a handful of notable exceptions, most studies describe the advantages of double lung transplants over single lung transplants in patients with pulmonary hypertension. However, this observation is not universal in the literature, and even studies that support DLT lack the clarity of consistent results.

The use of ECMO is often neglected in both arms of research, even though the negative sequelae of PAH after SLT may be worse than DLT. The utility of ECMO is likely underappreciated and may counterbalance some of the negative impacts observed following single lung procedures.

Even more frustrating is the possibility that the recipients of single and double lung transplants may not even be comparable populations. Selection and publication biases neglect age, co-morbid conditions, clinical features, or the variability in resources, policies, and surgeon judgment.

The question is not whether DLT is better at resolving lung disease. Instead, we must ask if SLT is an acceptable form of therapy in a select group of high-risk patients. Further research should focus on how best to identify recipients that may benefit from each type of procedure, as well as the clinical utility of perioperative VA ECMO. With limited organ availability, we should not disregard any potential tool, especially when a single donor may provide relief to multiple recipients.

## Data Availability

Not applicable.
